# Mortality risk following self‐harm in young people: a population cohort study using the Northern Ireland Registry of Self‐Harm

**DOI:** 10.1111/jcpp.13784

**Published:** 2023-03-16

**Authors:** Emma Ross, Dermot O'Reilly, Denise O'Hagan, Aideen Maguire

**Affiliations:** ^1^ Centre for Public Health, Queen's University Belfast Belfast UK; ^2^ Public Health Agency Northern Ireland Belfast UK

**Keywords:** Self‐harm, suicide, epidemiology, adolescence, mental health

## Abstract

**Background:**

Self‐harm is a recognised predictor of suicide and is most common in those aged under 25 years. The aims of this study were to describe the characteristics of young people who present with self‐harm; quantify the risk of suicide and other causes of death during follow up, and to identify factors associated with mortality risk.

**Methods:**

The Northern Ireland Registry of Self‐Harm (NIRSH) is a national registry capturing complete data on all presentations made to the 12 Emergency Departments (EDs) in Northern Ireland (NI). Data relating to self‐harm presentations registered in the NIRSH between 2012 and 2015 were linked to primary care registrations and death records up until 31st December 2018. Logistic regression was employed to examine the factors associated with self‐harm. Cox regression was used to estimate mortality risk following self‐harm and explore the associated risk factors.

**Results:**

The cohort consisted of 390,740 individuals aged 10–24 years registered with a General Practitioner (GP) in NI. During follow‐up, 4,450 individuals presented with self‐harm. Rates of self‐harm were highest in females, those aged 20–24 years (OR_adj_ = 3.53, 95% CI 3.28–3.80, *p* < .001), and in the most deprived areas (OR_adj_ = 2.71, 95% CI 2.45–2.99, *p* < .001). Thirty five individuals who presented with self‐harm died by suicide, accounting for 23% of all suicide deaths in the cohort. Suicide risk was increased 19‐fold in those who presented with self‐harm after adjustment for age, sex and area‐level factors (HR_adj_ = 19.00, 95% CI 12.80–28.21, *p* < .001). Increased suicide risk was observed in males (HR_adj_ = 2.04, 95% CI 0.99–4.23, *p* = .05) and those using more violent methods of self‐injury (HR_adj_ = 3.89, 95% CI 1.65–9.13, *p* < .001).

**Conclusions:**

Young people who self‐harm are at a significantly greater risk of suicide. Almost a quarter of young people who died by suicide in NI had presented to EDs with self‐harm, highlighting that the ED may provide a nodal point of intervention among a typically hard to identify and reach population.

## Introduction

Globally, it is estimated that more than 120,000 young people aged under 25 years die from self‐inflicted causes each year, although this figure is likely a substantial underestimate given the frequent misclassification of suicide deaths as accidental causes (Global Burden of Disease Collaborative Network, 2020; Snowdon & Choi, 2020). Nonfatal self‐harm is recognised as one of the strongest risk factors for suicide and is most prevalent in people under the age of 25 years (Carroll, Metcalfe, & Gunnell, 2014; Favril, Yu, Uyar, Sharpe, & Fazel, 2022; Hawton, Saunders, & O'Connor, 2012; Olfson et al., 2018; Public Health Agency, 2019; Ribeiro et al., 2016). True estimates of self‐harm vary substantially due to differences in study design, self‐report biases and the classification systems used to define self‐harm (Gillies et al., 2018; Lim et al., 2019; Mars et al., 2016; Muehlenkamp, Claes, Havertape, & Plener, 2012). Nevertheless, community studies suggest that the lifetime risk of self‐harm in adolescents across the United Kingdom is between 10 and 18% (Geulayov et al., 2018; Morey, Mellon, Dailami, Verne, & Tapp, 2017; O'Connor, Rasmussen, & Hawton, 2014). The available evidence also indicates that self‐harm is a marked risk factor for suicide in young people, with relative risks ranging from 17 to 30 (Cybulski et al., 2022; Finkelstein et al., 2015; Hawton et al., 2020; Morgan et al., 2017; Olfson et al., 2018). In a further study, 54% of children and young people who died by suicide in England had a previous history of self‐harm (Rodway et al., 2016). However, many of these studies are based on a few mainly urban settings raising some uncertainty about the representativeness of the findings.

Although our understanding of suicidality has improved in recent years, much less has been reported on the risk factors among younger people. The prevalence of self‐harm is disproportionately high in young people, and given their neurodevelopmental, social and behavioural vulnerabilities, it is possible that their risk profiles differ from their adult counterparts (Beckman et al., 2018; Carroll et al., 2014; Chan et al., 2016; Harris, Beese, & Moore, 2019). Research suggests that suicide risk in young people who self‐harm is increased in males, and those with adverse childhood experiences, family adversity or a history of psychiatric disorders (Beckman et al., 2018; Cybulski et al., 2022; Hawton et al., 2020; Miranda‐Mendizabal et al., 2019; Ohlis et al., 2020). Suicide risk has also been linked to increased access to means and to method of self‐harm, with marked increases in suicide in those who use arguably more violent methods of harm such as hanging or asphyxiation (Hawton et al., 2012, 2020; Miranda‐Mendizabal et al., 2019).

While our understanding of the association between self‐harm and suicide risk in young people has been greatly expanded in light of the growing number of population‐based cohort studies (Beckman et al., 2018; Geulayov et al., 2018; Hawton et al., 2020; Hawton, Bergen, et al., 2012; Morgan et al., 2017; Ohlis et al., 2020), there remain concerns about the representativeness and generalisability of some of these findings. In particular, much of the current evidence in the UK is derived from the Multicentre Study of Self‐Harm in England (Geulayov et al., 2018; Hawton et al., 2020; Hawton, Bergen, et al., 2012). Although an important resource, the data captured within the Multicentre Study of Self‐Harm are collected from just five hospital sites in England, in the large urban epicentres of Oxford, Manchester and Derby. Given the strong association between urban living and risk of self‐harm and suicidality across the UK, it is unclear whether these findings are fully representative (Satherley, Hazell, Jones, & Hanna, 2022). Additionally, previous attempts to quantify the mortality risk associated with self‐harm relative to the wider population of young people with no history of self‐harm have relied on the use of expected population mortality estimates (Hawton et al., 2020). As such, these estimates do not account for important determinants of suicide risk such as settlement band and deprivation, at the level of the individual. Further studies linking complete, national self‐harm data to individual‐level mortality data for both the exposed and unexposed populations are, therefore, warranted to further our understanding of the factors which place young people at an increased risk of suicide following self‐harm.

Northern Ireland (NI) is unique within the UK in that it holds a national registry of individuals who present to all 12 national hospital emergency departments (EDs) with self‐harm. NI has the highest rate of self‐harm in the UK, with an incidence of 20.1 presentations per 10,000 people compared to 11.4 per 10,000 people in England, and a disproportionately higher prevalence of mental illness (Carr et al., 2016; Department of Health, 2020; O'Neill & O'Connor, 2020). Some of this increased psychiatric morbidity has been attributed to the transgenerational effects of civil conflict known colloquially as ‘The Troubles’ (McLafferty et al., 2018; O'Neill & O'Connor, 2020). Developing a greater understanding of the risk factors for self‐harm and suicide is of vital public health importance given that the early identification of vulnerable populations is instrumental in ensuring effective treatment pathways and reducing negative outcomes.

This study utilises complete national data on all presentations made to the 12 EDs in NI to explore the association between ED‐presenting self‐harm and mortality risk among the whole population of young people in NI. The aims of the study were to describe the characteristics of young people who present with self‐harm in NI; quantify the risk of suicide and other causes of death during follow up, and to identify among those presenting with self‐harm the factors associated with increased mortality risk. Although suicide is likely to represent the majority of excess deaths in those presenting with self‐harm, we include both deaths from external causes and all‐cause mortality, as previous studies have identified higher risks of other causes of death in young people who self‐harm (Finkelstein et al., 2015; Hawton et al., 2020; King et al., 2019; Morgan et al., 2017).

## Methods

### Study design and setting

The Northern Ireland Registry of Self‐Harm (NIRSH) which gathers, validates and collates information on self‐harm presentations to all 12 EDs nationwide, has been operational since April 2012. Data are extracted from ED records by trained data collectors using standardised criteria and include all episodes of intentional self‐harm regardless of suicidal intent or motivation, and where the individual was alive upon arrival at the ED. Accidental harm, and acts of self‐harm carried out by individuals with a learning disability are excluded. Data captured included age at presentation, gender, date of presentation and method of self‐harm. To ensure sufficient statistical power, method of nonfatal self‐harm was aggregated into three categories: Sharp object (X78), self‐poisoning (X60‐X65) and other (X66‐X71, X76‐X84; See Table S1 for more detailed information on method of self‐harm). The patient's Health and Care Number (HCN), a unique 10‐digit number used throughout the Health Service in NI, is also captured; this enabled the identification of those who presented with more than one episode of self‐harm during the study, irrespective of ED of presentation.

For this study, data were extracted on all self‐harm presentations registered between 1st April 2012 and 31st December 2015 and linked to the national central health card registration system (National Health Application and Infrastructure Services (NHAIS) system) using the HCN as the linkage key. The NHAIS contains information on all patients registered with a primary care physician. NI has a universal, free at the point of service healthcare system so almost the entire population is registered. The NHAIS receives regular updates on date and cause of death from the General Register's Office, although there are known delays in defining cause of death with cases such as suicide that require coronial review (Northern Ireland Statistics and Research Agency, 2013). Death data were available until 31st December 2018, with suicides identified using ICD‐10 codes indicating intentional self‐harm (ICD10: X60‐X84) and sequalae of intentional self‐harm (ICD10: Y87.0). External deaths, including deaths due to accidents, assaults, intentional self‐harm and events of undetermined intent, were identified using codes V01‐Y98 of the ICD‐10 (See Table S2).

The study cohort consisted of all individuals aged 10–24 years (defined on 31st December 2015; *n* = 395,771) resident in NI between 1st April 2012 and 31st December 2015 who were registered with a General Practitioner (GP). This cohort was used to identify comparators who did not present with self‐harm and to define denominators for the calculation of self‐harm rates. Patient's address was used to append information about the characteristics of the area of residence. Urban/rural residence was based on a classification of settlements; urban (comprising the two largest cities), intermediate or rural (settlements with less than 2,250 people; Northern Ireland Statistics and Research Agency, 2005). Area deprivation was based on the NI Multiple Deprivation Measure 2010 and was divided into quintiles (Northern Ireland Statistics and Research Agency, 2010). There were 5,031 (1.3%) cohort members for whom area‐level information was missing. These individuals were excluded from the primary analysis due to small numbers which infringed on statistical disclosure regulations.

The project was designed in collaboration with the Self Harm Registry Steering Committee and approved by the HBS Governance Board. Ethical approval was granted by the Research Ethics Committee (REC) – REF 19/LO/1601. The HCN, and any other potentially identifiable service user data, was removed from the dataset prior to researcher access within the secure environment at the Honest Broker Service (HBS). All statistical outputs were subject to additional disclosure control measures, including restrictions on cell numbers to protect confidentiality.

### Statistical analysis

Descriptive statistics, followed by logistic regression, were employed to examine the characteristics of those presenting with self‐harm. Fully adjusted models included information on age, sex and area of residence. Interaction tests were employed to check for subgroup differences in the odds of presenting with self‐harm. The incidence of ED‐presenting self‐harm was calculated in person‐years, with further stratification by age and sex.

Separate Cox proportional hazards models were used to examine the risk of suicide, death by external causes, and all‐cause mortality in young people who presented with self‐harm compared to their peers in the general population, with follow up until 31st December 2018. Models examining cause‐specific mortality were censored at date of death for all causes to account for competing risks. Interaction tests were used to identify the presence of any statistically significant subgroup differences in the risk of all‐cause and cause‐specific mortality. Incidence of all‐cause mortality, death by external causes and suicide per 100,000 person‐years were calculated and stratified by self‐harm status.

Analyses were repeated on the subgroup of individuals who presented with self‐harm to identify socio‐demographic and presentation‐specific factors related to risk of death following self‐harm. Individuals were followed up from the date of first presentation to either death, or the end of follow‐up as defined above. The length of follow‐up ranged from 1 month to 6 years and 9 months. Overall, the mean follow‐up period was 4 years and 8 months. Incidence of all‐cause mortality, death by external causes and suicide per 100,000 person‐years were calculated, and further stratified by year of follow‐up to examine the relationship between mortality risk and time of presentation. Analyses were conducted using STATA 15.

## Results

### Study population

The final cohort consisted of 390,740 individuals aged 10–24 years. Between 1st April 2012 and 31st December 2015, there were 7,889 self‐harm presentations by 4,450 cohort members. This represented 1.14% of the cohort and equated to an incidence rate of 306 per 100,000 person‐years. Stratification by age and sex indicated that the incidence of self‐harm in young women peaked between 18 and 19 years at 578 per 100,000 person‐years, whilst the peak incidence for males occurred between 20–24 years at 516 per 100,000 person‐years. Over half (58.0%) of self‐harm presenters were female and approximately 30% had two or more presentations during the study period.

Overall, the odds of presenting with self‐harm were lower in males (OR_adj_ = 0.71 95% CI 0.67–0.76, *p* < .001), and higher in those living in urban areas (OR_adj_ = 1.83, 95% CI 1.67–2.01, *p* < .001), and in the most deprived areas (OR_adj_ = 2.71, 95% CI 2.45–2.99, *p* < .001; Table S3). Further analysis confirmed a significant age/sex interaction (*p* < .001), therefore, the logistic regression model was stratified by sex (Table 1). The ORs of presenting with self‐harm for those aged 18–19 years compared to those under 18 years were OR = 5.93, 95% CI 5.06–6.96, *p* < .001 for males, and OR = 2.63, 95% CI 2.36–2.91, *p* < .001 for females. For those aged 20–24 years compared to those aged under 18 years, the ORs were OR = 8.30, 95% CI 7.23–9.52, *p* < .001 for males, and OR = 2.14, 95% CI 1.96–2.35, *p* < .001 for females. Area of residence was also important and the odds of presenting with self‐harm increased stepwise as the degree of area‐level deprivation and population density increased, so that more than one third of self‐harm cases came from the most deprived quintile of the population, and almost half came from the most urban areas (Table S3). There was some evidence that area‐level characteristics had a greater influence on male self‐harm presentations; males in the most deprived areas were 3.5 times more likely to present with self‐harm than those in the most affluent areas (OR_adj_ = 3.48, 95% CI 2.98–4.07, *p* < .001), and those in urban areas were approximately twice as likely to present compared to their rural counterparts (OR_adj_ = 1.95, 95% CI 1.68–2.26, *p* < .001). In comparison, the odds of presenting with self‐harm among females in the most deprived areas were increased 2‐fold (OR_adj_ = 2.23, 95% CI 1.96–2.54, *p* < .001), while a 1.8‐fold increase was observed for those in urban areas (OR_adj_ = 1.78, 95% CI 1.58–2.00, *p* < .001).

**Table 1:** Logistic regression models demonstrating the odds of presenting to the emergency department with self‐harm given socio‐demographic characteristics, stratified by sex. Figures represent odds ratios (95% confidence intervals)

	Males			Females		
	No. presenting with self‐harm (% col)	Unadjusted OR (95% CI)	Fully adjusted OR^a^ (95% CI)	No. presenting with self‐harm (% col)	Unadjusted OR (95% CI)	Fully adjusted OR^a^ (95% CI)
Age (years)						
10–17	245 (13.1)	1.00	1.00	824 (31.9)	1.00	1.00
18–19	399 (21.3)	5.88 (5.01–6.90)**	5.93 (5.06–6.96)**	618 (24.0)	2.62 (2.36–2.91)**	2.63 (2.36–2.91)**
20–24	1,226 (65.6)	8.30 (7.23–9.52)**	8.30 (7.23–9.52)**	1,138 (44.1)	2.16 (1.98–2.37)**	2.14 (1.96–2.35)**
Area‐level deprivation						
Least deprived	204 (10.9)	1.00	1.00	329 (12.8)	1.00	1.00
Less deprived	264 (14.1)	1.17 (0.98–1.41)	1.42 (1.18–1.72)**	454 (17.6)	1.27 (1.10–1.47)**	1.52 (1.31–1.75)**
Intermediate	253 (13.5)	1.16 (0.96–1.39)	1.47 (1.22–1.78)**	435 (16.9)	1.25 (1.08–1.44)**	1.53 (1.32–1.77)**
More deprived	387 (20.7)	1.77 (1.50–2.10)**	2.06 (1.73–2.45)**	559 (21.7)	1.61 (1.40–1.85)**	1.86 (1.62–2.15)**
Most deprived	762 (40.7)	3.51 (3.01–4.10)**	3.48 (2.98–4.07)**	803 (31.1)	2.28 (2.01–2.60)**	2.23 (1.96–2.54)**
Settlement band						
Rural	269 (14.4)	1.00	1.00	473 (18.3)	1.00	1.00
Intermediate	683 (36.5)	2.21 (1.92–2.54)**	1.80 (1.55–2.08)**	893 (34.6)	1.63 (1.45–1.82)**	1.50 (1.34–1.69)**
Urban	918 (49.1)	2.62 (2.29–3.01)**	1.95 (1.68–2.26)**	1,214 (47.1)	1.92 (1.73–2.14)**	1.78 (1.58–2.00)**

^a^ Adjusted for age, area‐level deprivation and settlement band.

** *p* < .01.

Self‐poisoning was the most common method of self‐harm (68.8%), followed by self‐injury with a sharp object (24.0%), and other methods of self‐harm (7.4%; Figures available in Table 3). There were no recorded incidences of nonfatal intentional self‐harm by firearm. Table S1 provides more detailed information on self‐harm methods. Self‐poisoning was more common among females (72.2% vs. 63.6%) while other more violent methods of self‐harm such as hanging, strangulation or suffocation were more common among males (11.5% vs. 4.4%). The proportion of those presenting with self‐cutting was comparable in males and females (24.9% vs. 23.5%). There was little variation in method of self‐harm by age, although the proportion of those using more violent methods increased slightly with increasing age (6.6% in those aged 10–17 years; 7.8% in those aged 18–19 years; and 8.2% in those aged 20–24 years). Self‐cutting was somewhat more prevalent in urban residents compared to those from rural areas (25.2% vs. 22.1%), while a marginally higher proportion of rural dwellers used more violent methods of self‐harm compared to urban residents (9.7% vs. 7.1%; Table S4).

### Mortality risk associated with self‐harm

**Figure 1:**
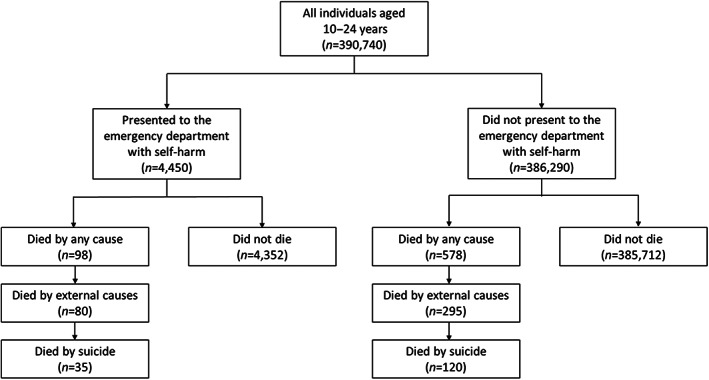
Flow chart showing numbers and causes of death within the subgroups of those who did and did not present with self‐harm

Overall, 676 cohort members died during follow‐up, 98 (14.5%) of whom had presented with self‐harm between 1st April 2012 and 31st December 2015 (Figure 1). Table S5 provides an overview of the socio‐demographic characteristics of those who died, stratified by self‐harm status.

There were 375 deaths from external causes including 155 suicides, of which 21.3% and 22.6%, respectively, were in individuals who self‐harmed (Table 2). Over a third (*n* = 35/98) of all deaths observed in those who presented with self‐harm were attributable to suicide.

**Table 2:** Cox proportional hazards models investigating the risk of all‐cause mortality, external deaths and self‐inflicted deaths associated with presentation to emergency departments with self‐harm

	Died (% col)	Unadjusted HR (95% CI)	Adjusted HR^a^ (95% CI)
**All‐cause (*N* = 676)**			
Self‐harm			
No	578 (85.5)	1.00	1.00
Yes	98 (14.5)	14.90 (12.00–18.41)**	11.97 (9.60–14.93)**
**External deaths (*N* = 375)**			
Self‐harm			
No	295 (78.7)	1.00	1.00
Yes	80 (21.3)	23.78 (18.57–30.44)**	17.20 (13.30–22.26)**
**Self‐inflicted deaths (*N* = 155)**			
Self‐harm			
No	120 (77.4)	1.00	1.00
Yes	35 (22.6)	25.57 (17.55–37.26)**	19.00 (12.80–28.21)**

^a^ Adjusted for age, sex, area‐level deprivation and settlement band.

** *p* < .01.

The incidence of suicide during follow‐up was 118 per 100,000 persons‐years in those who presented with self‐harm compared to 5 per 100,000 person‐years in those with no record of ED‐presenting self‐harm. There was evidence that the risk of suicide among self‐harmers was greatest in the months most proximal to self‐harm presentation, with approximately 37% of all suicide deaths occurring within the first 12 months following index self‐harm presentation (Table S6). This was reflected upon examination of yearly incidence rates; the incidence of suicide was 293 per 100,000 person‐years in the first 12 months following index presentation, decreasing to 226 per 100,000 person‐years in the second year and 100 per 100,000 person‐years in the third year following self‐harm. Similar attenuations in risk were observed for both external causes of death and all‐cause mortality (Table S6).

In unadjusted models, the risk of suicide in those presenting with self‐harm was over 25 times (HR 25.57 95%CI 17.55–37.26, *p* < .001) that of those who did not present with self‐harm (Table 2), although with further adjustment for socio‐demographic characteristics, this was reduced to HR_adj_ = 19.00 (95% CI 12.80–28.21, *p* < .001). Individuals who self‐harmed also had an elevated risk of death due to external causes HR_adj_ = 17.20 (95% CI 13.30–22.26, *p* < .001) and from all causes HR_adj_ = 11.97 (95% CI 9.60–14.93, *p* < .001). Among those who died by suicide, there was little variation in the socio‐demographic characteristics of those who presented with self‐harm compared to those who did not present with self‐harm. Suicide decedents who presented with self‐harm were generally older than their counterparts who did not present with self‐harm (χ^2^ = 7.0794, *p* = .029). There was also some evidence of a higher proportion of female suicide decedents and a higher proportion of decedents from urban areas among self‐harmers, however, these differences were not statistically significant.

### Death by suicide and external causes following self‐harm

Table 3 shows the relationship between socio‐demographic and presentation‐specific factors and risk of death by suicide, external causes and all causes.

**Table 3:** Cox Proportional Hazard models investigating the risk factors associated with all‐cause mortality, death by external causes and death due to self‐inflicted injuries following presentation with self‐harm

	Presented with self‐harm (% col)	All‐cause mortality (*N* = 98)		External death (*N* = 80)		Self‐inflicted death (*N* = 35)	
		Unadjusted HR (95% CI)	Adjusted HR^a^ (95% CI)	Unadjusted HR (95% CI)	Adjusted HR^a^ (95% CI)	Unadjusted HR (95% CI)	Adjusted HR^a^ (95% CI)
Sex							
Female	2,580 (58.0)	1.00	1.00	1.00	1.00	1.00	1.00
Male	1,870 (42.0)	3.53 (2.28–5.47)**	2.89 (1.83–4.57)**	3.97 (2.41–6.53)**	3.16 (1.88–5.31)**	2.36 (1.19–4.68)*	2.04 (0.99–4.23)
Age at presentation (years)							
10–17	1,069 (24.0)	1.00	1.00	1.00	1.00	1.00	1.00
18–19	1,017 (22.9)	2.03 (1.26–3.26)*	1.61 (0.99–2.62)	1.95 (1.14–3.33)*	1.52 (0.88–2.63)	0.95 (0.41–2.17)	0.80 (0.34–1.86)
20–24	2,364 (53.1)	1.80 (1.08–3.01)*	1.39 (0.82–2.35)	2.03 (1.16–3.53)*	1.54 (0.87–2.73)	1.30 (0.60–2.83)	1.08 (0.48–2.43)
Area‐level deprivation							
Least deprived	533 (12.0)	1.00	1.00	1.00	1.00	1.00	1.00
Less deprived	718 (16.1)	0.61 (0.25–1.47)	0.62 (0.25–1.51)	0.50 (0.18–1.40)	0.51 (0.18–1.46)	0.75 (0.22–2.57)	0.83 (0.24–2.94)
Intermediate	688 (15.5)	0.92 (0.41–2.05)	0.87 (0.38–1.97)	0.95 (0.39–2.29)	0.87 (0.36–2.14)	0.47 (0.11–1.95)	0.47 (0.11–2.00)
More deprived	946 (21.3)	0.93 (0.44–1.96)	0.85 (0.40–1.82)	1.13 (0.51–2.52)	1.07 (0.48–2.41)	1.13 (0.39–3.32)	1.22 (0.41–3.63)
Most deprived	1,565 (35.2)	1.48 (0.77–2.85)	1.20 (0.62–2.34)	1.38 (0.67–2.87)	1.10 (0.52–2.30)	0.82 (0.29–2.34)	0.68 (0.23–1.95)
Settlement band							
Rural	742 (16.7)	1.00	1.00	1.00	1.00	1.00	1.00
Intermediate	1,576 (35.4)	1.34 (0.70–2.60)	1.11 (0.56–2.20)	1.12 (0.55–2.27)	0.93 (0.44–1.93)	1.04 (0.36–3.00)	0.97 (0.33–2.88)
Urban	2,132 (47.9)	1.50 (0.80–2.81)	1.02 (0.51–2.05)	1.35 (0.70–2.62)	0.97 (0.46–2.02)	1.32 (0.49–3.54)	1.34 (0.46–3.91)
Number of presentations							
One	3,127 (70.3)	1.00	1.00	1.00	1.00	1.00	1.00
Two+	1,323 (29.7)	2.28 (1.53–3.40)**	2.16 (1.45–3.24)**	2.71 (1.74–4.22)**	2.61 (1.66–4.10)**	1.49 (0.76–2.93)	1.49 (0.75–2.96)
Method of self‐harm							
Self‐poisoning	3,053 (68.8)	1.00	1.00	1.00	1.00	1.00	1.00
Sharp object	1,069 (24.0)	1.28 (0.81–2.02)	1.13 (0.72–1.79)	1.42 (0.86–2.35)	1.23 (0.74–2.03)	1.43 (0.64–3.18)	1.34 (0.60–2.99)
Other	328 (7.4)	1.89 (0.99–3.59)	1.49 (0.78–2.86)	2.46 (1.28–4.76)**	1.92 (0.98–3.74)	4.38 (1.90–10.08)**	3.89 (1.65–9.13)**

^a^ Adjusted for sex, age at presentation (years), area‐level deprivation, settlement band, number of presentations and method of self‐harm.

* *p* < .05.

** *p* < .01.

There was little evidence of variation in suicide risk by age at presentation. Males were approximately twice as likely to die by suicide (HR = 2.36, 95% CI 1.19–4.68, *p* = .014), although this was attenuated with adjustment for method of self‐harm to HR_adj_ = 2.04, 95% CI 0.99–4.23, *p* = .05. Area‐level deprivation, although associated with an increased risk of presenting with self‐harm, was not associated with an increased risk of suicide following presentation. Additionally, there were no statistically significant differences in suicide risk for those residing in urban areas (HR_adj_ = 1.34, 95% CI 0.46–3.91, *p* = .97) or those with multiple self‐harm presentations (HR_adj_ = 1.49, 95% CI 0.75–2.96, *p* = .26). The risk of suicide was approximately four times higher in those who self‐harmed using more violent methods compared to those who self‐poisoned (HR_adj_ = 3.89, 95% CI 1.65–9.13, *p* < .001). While those who self‐cut also appeared to be at an increased risk of suicide, this association was not statistically significant in either the unadjusted (HR = 1.43, 95% CI 0.64–3.18, *p* = .38) or fully‐adjusted analyses (HR_adj_ = 1.34, 95% CI 0.60–2.99, *p* = .48). Although self‐poisoning accounted for the greatest proportion of ED presentations for self‐harm (69%), the overwhelming majority of suicides identified by the Coroner were by intentional hanging, strangulation or suffocation. There were no deaths resulting from intentional self‐harm by firearm.

There were 80 deaths by external causes, 35 of which were due to intentional self‐inflicted injury. Further examination by cause of external death revealed that approximately half were classified as deaths of undetermined intent by the Coroner. Self‐poisonings of any intent, including intentional, undetermined and accidental self‐poisonings represented the leading cause of death among self‐harmers, ahead of intentional self‐injury by means other than self‐poisoning. Mortality from external deaths was highest for those aged over 18 years and in males (HR_adj_ = 3.16, 95% CI 1.88–5.31, *p* < .001; Table 3). Neither deprivation nor urban/rural residency were related to risk of death by external causes. Individuals with multiple self‐harm presentations experienced a 2.6‐fold increased risk of death by external causes compared to those with one presentation (HR_adj_ = 2.61, 95% CI 1.66–4.10, *p* < .001). There were some disparities in suicide method between single and multiple presenters, with the latter experiencing a significantly greater proportion of deaths due to self‐poisonings of undetermined intent.

There were only an additional 18 deaths due to nonexternal causes, so the patterns of risk factors associated with all‐cause and external factors were very similar. The main difference was an attenuation in the relative risks for the number of presentations and the methods of self‐harm.

## Discussion

To our knowledge, this is the first study to capture complete national data on all presentations made to EDs for self‐harm, enabling a fully representative examination of the association between ED‐presenting self‐harm and mortality risk among the whole population of young people in NI. The findings of this study indicate that adolescents who self‐harm have a significantly increased risk of mortality compared to their peers in the general population, particularly death due to suicide. We observed that 37% of deaths occurring in those who presented with self‐harm were a result of intentional self‐inflicted injuries, with more than a third occurring within the first 12 months following self‐harm.

This study confirms the high incidence of ED‐presenting self‐harm among young people in NI, demonstrating an overall incidence rate of 306 per 100,000 person‐years. Consistent with findings from previous studies, self‐harm was more prevalent among women, those from more socially deprived backgrounds and in larger conurbations where most of the EDs are situated (Cairns, Graham, & Bambra, 2017; Cybulski et al., 2022; Hawton et al., 2020; Hawton, Bergen, et al., 2012; Samaritans, 2017). This might indicate confounding as both ED attendance in general and presentations for self‐harm are known to be strongly influenced by proximity to hospitals (Giebel et al., 2019; Rudge et al., 2013). However, we observed minimal variation in lethality of self‐harm method at presentation or of subsequent mortality risk between urban and rural residents.

Although the majority of young people in this study who died had no record of ED‐presenting self‐harm, almost a quarter of those who died by suicide had previously presented to the ED with self‐harm, highlighting that the ED may provide a nodal point of intervention among a typically hard to identify and reach population. Self‐harm was a marked risk factor for suicide, even after adjustment for confounders, accounting for a 19‐fold increase in the risk of suicide compared to the general population of young people in NI. In a comparable ED‐based study in the UK, Hawton et al. observed a 30‐fold increase in the expected incidence of suicide in adolescents who self‐harmed (Hawton et al., 2020). Although this estimate is considerably higher than that observed in the current study, there are a number of plausible explanations for this disparity. Firstly, Hawton et al.'s estimate represents suicide risk in the first year following self‐harm. It is widely recognised, and evident in the current study, that suicide risk peaks in the months immediately following self‐harm (Geulayov et al., 2019). We observed that almost 40% of self‐inflicted deaths occurred within the first 12 months following self‐harm, representing the peak annual incidence of 293 per 100,000 person‐years. Furthermore, Hawton et al.'s study cohort was derived from five hospitals in the large conurbations of Manchester, Oxford and Derby. Urban residence is recognised as a significant risk factor for suicide, therefore, a comparatively larger risk estimate is to be expected in areas with predominantly urban catchment areas (Satherley et al., 2022). Additionally, these risk estimates were calculated using the expected population mortality, and as such, the authors were unable to adjust for important determinants of suicide risk such as settlement band and deprivation at the individual level, which in the current study, attenuated the risk estimate by approximately a quarter. In a cohort study examining self‐harm and subsequent mortality using primary care data, Morgan et al. (2017) observed a similar 17‐fold increase in the risk of suicide among self‐harmers. This lower estimate is not unexpected given the reduced severity of self‐harm episodes presenting to primary care providers. This rationale was somewhat exemplified by Cybulski et al. (2022) who utilised a case–control design to examine the risk factors for self‐harm and suicide in adolescents in England with indicators of self‐harm derived from multiple settings, including primary care, the ED, and hospital admissions data. Despite capturing a wider range of severity in self‐harm episodes, Cybulski et al. also reported a 19‐fold increase in the risk of suicide in adolescents with a history of self‐harm, therefore, lending its support to the representativeness of the current study.

Approximately three quarters of young people who died by suicide had no history of ED‐presenting self‐harm. Aside from our observation that suicide decedents who presented with self‐harm were generally older than their counterparts who did not present with self‐harm, there were no significant differences in the demographic profiles or indeed, of suicide method between these individuals. There is some evidence that the majority of those who die by suicide do so on their first attempt (Jordan & McNiel, 2020). While it is plausible that this may in part explain the high number of suicides observed among young people who did not present with self‐harm, it is also important to recognise that these individuals may have presented to an ED outside of the defined study period, or engaged in self‐harm that was not severe enough to require attendance at the ED.

We identified several risk factors for mortality following self‐harm. As with previous research in this area, the risk of suicide was higher in males and those who used more violent methods of self‐harm, such as hanging or drowning (Hawton et al., 2020; Hawton, Bergen, et al., 2012; Miranda‐Mendizabal et al., 2019; Morgan et al., 2017). Similar findings have been reported by Hawton et al. who recorded a 5‐fold increase in the risk of suicide in those who self‐harmed by hanging or asphyxiation, and Beckman et al. who observed an eight‐fold higher risk in adolescents who self‐harmed by more violent methods compared to those who self‐poisoned (Beckman et al., 2018; Hawton et al., 2020). The high lethality associated with these methods of self‐harm suggests an increased suicidal intent that is both immediate and enduring.

Initial comparison of method of self‐harm and method of suicide provided support for the escalation of lethality in method in those with suicidal intent (Finkelstein et al., 2015; Hawton et al., 2020; Hawton, Bergen, et al., 2012). While the overwhelming majority of presentations were due to self‐poisoning, over 80% of deaths identified as intentional self‐inflicted injuries by the Coroner occurred as a result of strangulation, hanging or asphyxiation. However, when deaths of undetermined or accidental intent were included in the analysis, self‐poisonings of any intent represented the leading cause of death overall. These findings, together with those from previous studies highlight that death from drug‐related substance misuse, whether intentional or accidental, is a significant and prevalent cause of death among young people who self‐harm (Hawton et al., 2020; King et al., 2019; Morgan et al., 2017; Ohlis et al., 2020). The challenges associated with determining intent of self‐poisonings are widely acknowledged, and it is recognised that suicides resulting from self‐poisoning are commonly misclassified as accidental poisonings or poisonings of undetermined intent (Snowdon & Choi, 2020). When these deaths were included, the majority who self‐harmed by self‐cutting or self‐poisoning died by self‐poisoning, while those who self‐harmed using more violent methods died by similar causes. It is important to note, however, that we examined risk of mortality from first recorded self‐harm presentation. It is, therefore, possible that those with multiple presentations switched to a more lethal form of self‐harm in episodes more proximal to death, possibly resulting from increased suicidal intent. Notably, there were no incidences of fatal or nonfatal intentional self‐harm by firearm in the current study. This finding heavily contrasts with observations from the United States where an estimated 51% of suicide deaths in young people involve a firearm (Centre for Disease Control and Prevention, 2021). This disparity likely reflects the significant between‐country variation in firearms licencing laws and gun culture, with NI having a considerably lower overall prevalence of firearm ownership resulting from strict firearms licencing legislation (Small Arms Survey, 2018).

While the proportion of those dying by suicide was greatest in those living in the most deprived areas, there was no evidence that this conveyed a greater risk of mortality among ED‐presenting self‐harmers. There is compelling evidence of an association between social disadvantage and suicide risk or self‐harm independently, but few studies have attempted to disentangle the relationship between social disadvantage and suicide risk among those who self‐harm (Cairns et al., 2017; Cybulski et al., 2022; Li, Page, Martin, & Taylor, 2011; Milner, Page, & Lamontagne, 2014; Samaritans, 2017). It is possible that the current study lacked sufficient power to reliably investigate this relationship, therefore, further studies with larger self‐harm cohorts are warranted.

This study offered an unparalleled opportunity to examine the association between validated ED‐presenting self‐harm and suicide risk on a population‐wide basis. The availability of a national, prospectively measured indicator of self‐harm enabled the examination of this association on a fully representative cohort, and surmounted many of the limitations of previous survey‐based studies which are subject to responder and recall biases. There are, however, some limitations to consider. As the study was restricted to ED‐presenting self‐harm, it is unclear whether these findings are generalisable to the wider body of young people in the community who self‐harm. This is especially pertinent given the widely acknowledged differences in community‐level and clinically presenting self‐harm, particularly regarding the method of self‐harm utilised and its associated severity (Hawton & Harriss, 2007; Madge et al., 2008). Future studies incorporating linkages to alternative sources of self‐harm data, for example, primary care data, are clearly warranted. It should be noted that information on psychiatric morbidity is not recorded in the Registry. As such, we were unable to examine the effect of potential confounding variables such as the presence of psychiatric disorders or indeed, the differential care pathways provided in the ED setting which may have influence over subsequent mortality risk. In line with the World Health Organisation's definition of self‐harm, the Registry captures episodes of self‐harm regardless of suicidal intent or motive. It was, therefore, not possible to differentiate between suicidal and nonsuicidal self‐harm. While studies from Sweden have indicated that self‐harm with evidence of suicidality appears to have a stronger association with repeat self‐harm and other adverse sequalae than nonsuicidal self‐harm (Bjureberg et al., 2019, 2022), evidence from the United States (Asarnow et al., 2011) and the United Kingdom (Wilkinson, Kelvin, Roberts, Dubicka, & Goodyer, 2011) confirm that nonsuicidal self‐harm is also a strong predictor of repeat self‐harm. This distinction, therefore, remains controversial as nonsuicidal self‐harm has strong and significant links to suicide and other suicidal behaviours. Additionally, it is difficult to reliably ascertain suicidal intent or motivation given the often ephemeral nature of suicidality, and the reluctance to disclose suicidal intent as a result of stigma and fear of unwanted hospitalisation (Deming et al., 2021). Nevertheless, the ED provides a critical opportunity to identify high‐risk individuals and deliver interventions to a typically clinically inaccessible group of individuals.

## Conclusions

Almost a quarter of young people who died by suicide in NI had presented to the ED with self‐harm before their death, highlighting the importance of tackling self‐harm in youth suicide prevention. Given the strong link between self‐harm and suicide, EDs provide an important setting in which to identify individuals at an increased risk of repeat self‐harm, and suicide, provide frontline mental health assessments, and to collaborate with relevant healthcare providers to ensure continuity of care in the community. Additionally, these findings highlight that death from drug‐related substance misuse, whether intentional or accidental, is a significant and prevalent cause of death among young people who self‐harm. Further work is required to examine the differential care pathway provided to individuals who present with self‐harm both at the time of presentation at the ED and in the postpresentation period and how this influences future suicide risk.

## Supporting information


**Table S1.** Methods of nonfatal self‐harm employed by cohort members.
**Table S2.** ICD‐10 codes identifying external causes of morbidity and mortality (V01‐Y98).
**Table S3.** Socio‐demographic characteristics of individuals in the cohort and odds ratios and 95% confidence intervals of presenting to emergency departments with self‐harm among socio‐demographic groups.
**Table S4.** Number of individuals presenting with self‐harm by settlement band and method of self‐harm.
**Table S5.** Socio‐demographic characteristics of individuals in the cohort who died, stratified by self‐harm status.
**Table S6.** Incidence of all‐cause mortality, death by external causes and death due to self‐inflicted injuries by number of years since self‐harm presentation per 100,000 person years (95% confidence intervals).
