# Supplementary material for: Mortality risk following self‐harm in young people: a population cohort study using the Northern Ireland Registry of Self‐Harm
Source: J Child Psychol Psychiatry. 2023 Mar 16;64(7):1015–26. doi: 10.1111/jcpp.13784 (PMC10952668; doi:10.1111/jcpp.13784)
Supplement: Supplementary file 1 — Table S1. Methods of nonfatal self‐harm employed by cohort members. Table S2. ICD‐10 codes identifying external causes of morbidity and mortality (V01‐Y98). Table S3. Socio‐demographic characteristics of individuals in the cohort and odds ratios and 95% confidence intervals of presenting to emergency departments with self‐harm among socio‐demographic groups. Table S4. Number of individuals presenting with self‐harm by settlement band and method of self‐harm. Table S5. Socio‐demographic characteristics of individuals in the cohort who died, stratified by self‐harm status. Table S6. Incidence of all‐cause mortality, death by external causes and death due to self‐inflicted injuries by number of years since self‐harm presentation per 100,000 person years (95% confidence intervals). [file JCPP-64-1015-s001.docx]

**Supporting Information**

**Table S1. Methods of non-fatal self-harm employed by cohort members**

*Other includes intentional self-harm by smoke, fire and flames (X76), steam, hot vapours and hot objects (X77), blunt object (X79), crashing of motor vehicle (X82), other specified means (X83), or unspecified means (X84).

There were no incidences of intentional self-harm by firearms (X72-X74) or by explosive material (X75).

| **Method of self-harm (ICD-10 codes)** | **Number (%)** |
| --- | --- |
| Self-poisoning with prescription medication (X60-X64) | 3,009 (67.6) |
| Self-poisoning by exposure to alcohol (X65) | 44 (1.0) |
| Self-poisoning by exposure to organic solvents, gases and vapours, or pesticides (X66-X69) | 37 (0.8) |
| Hanging, strangulation, or suffocation (X70) | 151 (3.4) |
| Drowning or submersion (X71) | 34 (0.8) |
| Sharp object (X78) | 1,069 (24.0) |
| Jumping from a high place (X80) | 44 (1.0) |
| Jumping or lying before a moving object (X81) | 22 (0.5) |
| Other* | 40 (0.9) |

**Table S2. ICD-10 codes identifying external causes of morbidity and mortality (V01-Y98)**

| **ICD-10 Code** | **Description** |
| --- | --- |
|  | |
| **V01-X59** | **Accidents** |
| V01-V99 | Transport Accidents |
| W00-W19 | Falls |
| W20-W49 | Exposure to inanimate mechanical forces |
| W50-W64 | Exposure to animate mechanical forces |
| W65-W74 | Accidental drowning and submersion |
| W75-W84 | Other accidental threats to breathing |
| W85-W99 | Exposure to electric current, radiation and extreme ambient air temperature and pressure |
| X00-X09 | Exposure to smoke, fire and flames |
| X10-X19 | Contact with heat and hot substances |
| X20-X29 | Contact with venomous animals and plants |
| X30-X39 | Exposure to forces of nature |
| X40-X49 | Accidental poisoning by and exposure to noxious substances |
| X50-X57 | Overexertion, travel and privation |
| X58-X59 | Accidental exposure to other and unspecified factors |
|  | |
| **X60-X84** | **Intentional self-harm** |
| X60-X69 | Intentional self-harm by poisoning |
| X70 | Intentional self-harm by hanging, strangulation and suffocation |
| X71 | Intentional self-harm by drowning and submersion |
| X72 | Intentional self-harm by handgun discharge |
| X73 | Intentional self-harm by rifle, shotgun and larger firearm discharge |
| X74 | Intentional self-harm by other and unspecified firearm discharge |
| X75 | Intentional self-harm by explosive material |
| X76 | Intentional self-harm by smoke, fire and flames |
| X77 | Intentional self-harm by steam, hot vapours and hot objects |
| X78 | Intentional self-harm by sharp object |
| X79 | Intentional self-harm by blunt object |
| X80 | Intentional self-harm by jumping from a high place |
| X81 | Intentional self-harm by jumping or lying before moving object |
| X82 | Intentional self-harm by crashing of motor vehicle |
| X83 | Intentional self-harm by other specified means |
| X84 | Intentional self-harm by unspecified means |
|  | |
| **X85 – Y09** | **Assault** |
|  | |
| **Y10-34** | **Events of undetermined intent** |
| Y10-Y19 | Poisoning, undetermined intent |
| Y20 | Hanging, strangulation and suffocation, undetermined intent |
| Y21 | Drowning and submersion, undetermined intent |
| Y22 | Handgun discharge, undetermined intent |
| Y23 | Rifle, shotgun and larger firearm discharge, undetermined intent |
| Y24 | Other and unspecified firearm discharge, undetermined intent |
| Y25 | Contact with explosive material, undetermined intent |
| Y26 | Exposure to smoke, fire and flames, undetermined intent |
| Y27 | Contact with steam, hot vapours and hot objects, undetermined intent |
| Y28 | Contact with sharp object, undetermined intent |
| Y29 | Contact with blunt object, undetermined intent |
| Y30 | Falling, jumping or pushed from a high place, undetermined intent |
| Y31 | Falling, lying or running before or into moving object, undetermined intent |
| Y32 | Crashing of motor vehicle, undetermined intent |
| Y33 | Other specified events, undetermined intent |
| Y34 | Unspecified event, undetermined intent |
|  |  |
| Y35-Y36 | Legal intervention and operations of war |
|  | |
| **Y40-Y84** | **Complications of medical and surgical care** |
|  | |
| **Y85-Y89** | **Sequalae of external causes of morbidity and mortality** |
| Y85 | Sequelae of transport accidents |
| Y86 | Sequelae of other accidents |
| Y87 | Sequelae of intentional self-harm, assault and events of undetermined intent |
| Y87.0 | Sequelae of intentional self-harm |
| Y87.1 | Sequelae of assault |
| Y87.2 | Sequelae of events of undetermined intent |
| Y88 | Sequelae with surgical and medical care as external cause |
| Y89 | Sequalae of other external causes |
|  | |
| **Y90-Y98** | **Supplementary factors related to causes of morbidity and mortality classified elsewhere** |
|  | |

Code derived from International Statistical Classification of Diseases and Related Health Problems 10th Revision (ICD-10)

Suicide deaths were identified using codes X60-X84, and Y87.0

**Table S3. Socio-demographic characteristics of individuals in the cohort and odds ratios and 95% confidence intervals of presenting to emergency departments with self-harm among socio-demographic groups.**

|  | | Total population (% pop) | No. presenting with self-harm (% col) | Unadjusted OR (95% CI) | Fully-adjusted OR^a^ (95% CI) |
| --- | --- | --- | --- | --- | --- |
|  | | | | | |
| Sex | Female | 192,578 (49.3) | 2,580 (58.0) | 1.00 | 1.00 |
|  | Male | 198,162 (50.7) | 1,870 (42.0) | 0.70 (0.66 – 0.74)** | 0.71 (0.67 – 0.76)** |
|  | | | | | |
| Age (years) | 10-17 | 206,520 (52.9) | 1,105 (24.8) | 1.00 | 1.00 |
|  | 18-19 | 55,925 (14.3) | 981 (22.0) | 3.38 (3.10 – 3.68)** | 3.39 (3.11 – 3.69)** |
|  | 20-24 | 128,295 (32.8) | 2,364 (53.1) | 3.57 (3.32 – 3.84)** | 3.53 (3.28 – 3.80)** |
|  | | | | | |
| Area-level deprivation | Least deprived | 73,545 (18.8) | 533 (12.0) | 1.00 | 1.00 |
|  | Less deprived | 80,656 (20.6) | 718 (16.1) | 1.23 (1.10 – 1.38)** | 1.48 (1.32 – 1.66)** |
|  | Intermediate | 78,572 (20.1) | 688 (15.5) | 1.21 (1.08 – 1.36)** | 1.51 (1.34 – 1.69)** |
|  | More deprived | 78,537 (20.1) | 946 (21.3) | 1.67 (1.50 – 1.86)** | 1.94 (1.74 – 2.16)** |
|  | Most deprived | 79,430 (20.3) | 1,565 (35.2) | 2.75 (2.49 – 3.04)** | 2.71 (2.45 – 2.99)** |
|  | | | | | |
| Settlement band | Rural | 112,006 (28.7) | 742 (16.7) | 1.00 | 1.00 |
|  | Intermediate | 130,111 (33.3) | 1,576 (35.4) | 1.84 (1.68 – 2.01)** | 1.61 (1.47 – 1.76)** |
|  | Urban | 148,623 (38.0) | 2,132 (47.9) | 2.18 (2.01 – 2.37)** | 1.83 (1.67 – 2.01)** |

^a^adjusted for age, sex, area-level deprivation, and settlement band

*p<0.05

**p<0.01

**Table S4. Number of individuals presenting with self-harm by settlement band and method of self-harm.**

|  | | Settlement Band | | |
| --- | --- | --- | --- | --- |
|  |  | Urban  (N=2,132)  % col | Intermediate (N=1,576)  % col | Rural  (N=742)  % col |
|  | | | | |
| Method of Self-Harm | Sharp Object | 537 (25.2) | 368 (23.4) | 164 (22.1) |
|  | Self-Poisoning | 1,444 (67.7) | 1,103 (70.0) | 506 (68.2) |
|  | Other | 151 (7.1) | 105 (6.7) | 72 (9.7) |
|  | | | | |

**Table S5. Socio-demographic characteristics of individuals in the cohort who died, stratified by self-harm status**

|  | | Did not present with self-harm (N=578) | Presented with self-harm  (N=98) |
| --- | --- | --- | --- |
|  | | | |
| Sex | Female | 174 (30.1) | 28 (28.6) |
|  | Male | 404 (69.9) | 70 (71.4) |
|  | | | |
| Age (years) | 10-17 | 191 (33.0) | * |
|  | 18-19 | 96 (16.6) | * |
|  | 20-24 | 291 (50.4) | 70 (71.4) |
|  | | | |
| Area-level deprivation | Least deprived | 299 (51.7) | 33 (33.7) |
|  | Most deprived | 279 (48.3) | 65 (66.3) |
|  | | | |
| Settlement band | Rural | 183 (31.7) | 12 (12.2) |
|  | Intermediate | 191 (33.0) | 34 (34.7) |
|  | Urban | 204 (35.3) | 52 (53.1) |
|  | | | |
| Number of presentations | One | n/a | 47 (48.0) |
|  | Two+ | n/a | 51 (52.0) |
|  | | | |
| Method of self-harm | Self-poisoning | n/a | 60 (61.2) |
|  | Sharp object | n/a | 27 (27.6) |
|  | Other | n/a | 11 (11.2) |

*Cell values suppressed to conform with the principles of statistical disclosure control

**Table S6. Incidence of all-cause mortality, death by external causes and death due to self-inflicted injuries by number of years since self-harm presentation per 100,000 person years (95% confidence intervals)**

|  | All-cause mortality (N=98) | | External death (N=80) | | Self-inflicted death (N=35) | |
| --- | --- | --- | --- | --- | --- | --- |
|  | Number of deaths  (% col) | Incidence rate | Number of deaths  (% col) | Incidence rate | Number of deaths  (% col) | Incidence rate |
| 12 months | 26 (26.5) | 587 (399 – 861) | * | 451 (291 – 699) | 13 (37.1) | 293 (170 – 505) |
| 2 years | * | 408 (257 – 648) | * | 408 (257 – 648) | 10 (28.6) | 227 (122 – 421) |
| 3 years+ | * | 448 (343 – 585) | * | 342 (257 – 471) | 12 (34.3) | 100 (57 – 175) |

*Cell values suppressed to conform with the principles of statistical disclosure control
